# Improving the microbial sampling and analysis of secondary infected root canals by passive ultrasonic irrigation

**DOI:** 10.1007/s00784-022-04424-x

**Published:** 2022-02-26

**Authors:** Milena Kluge, Johanna Trüschler, Fadil Elamin, Annette Anderson, Elmar Hellwig, Markus Altenburger, Kirstin Vach, Annette Wittmer, Ali Al-Ahmad

**Affiliations:** 1 Department of Orthodontics and Dentofacial Orthopaedics, Dental School, University of Heidelberg, Im Neuenheimer Feld 400, 69120 Heidelberg, Germany; 2 Department of Operative Dentistry and Periodontology, Center for Dental Medicine, Medical Center, Faculty of Medicine, University of Freiburg, Hugstetter Strasse 55, 79106 Freiburg, Germany; 3 Khartoum Center for Research and Medical Training, Khartoum, Sudan; 4 Department of Medical Biometry and Statistics, Medical Center, Faculty of Medicine, University of Freiburg, Stefan-Meier-Strasse 26, 79104 D-Freiburg Im Breisgau, Germany; 5 Department of Microbiology and Hygiene, Institute of Medical Microbiology and Hygiene, Medical Center, Faculty of Medicine, University of Freiburg, Hermann-Herder-Strasse 11, 79104 D-Freiburg Im Breisgau, Germany

**Keywords:** PUI, Endodontic retreatment, Microbial sampling, Root canal treatment, Culture technique, PCR

## Abstract

**Objective:**

The persistence of pathogenic microorganisms in root canals is the most common reason for the failure of root canal treatment and the necessity of a root filling treatment, which results in an uncertain prognosis due to technical complexity and the variety of highly adaptable microorganisms.

This study evaluated the effect of passive ultrasonic irrigation (PUI) on the outcome of the microbial analysis of root canal-treated teeth with persistent or recurrent apical inflammation in vivo.

**Materials and methods:**

Sample collection was performed after root filling removal (sample S1, control group) and after PUI with NaCl (sample S2) using sterile paper points. In total, 19 samples were obtained. Quantification was performed by means of serial dilution of the samples. Subcultivated pure cultures were identified using MALDI-TOF MS complemented by the Vitek-2-System or PCR, followed by sequencing of the 16S rRNA gene. The results of the samples (S1 and S2) were evaluated regarding their bacterial count and composition.

**Results:**

The total count of bacteria and the number of aerobic/facultative anaerobic microorganisms significantly increased in the S2-samples after application of PUI. The number of obligate anaerobic microorganisms showed an increase after PUI, although it was not significant. We detected 12 different aerobic/facultative anaerobic microorganisms before PUI, and in 21 cases after PUI. Two different obligate anaerobic microorganisms were found in S1 samples compared to nine different species in S2 samples.

**Conclusions:**

PUI is a powerful method for detaching bacteria in infected root canals and enables a more precise analysis of the etiology of persistent endodontic infections.

**Clinical relevance:**

This study indicates that PUI exerts a positive cleansing effect and adds to the accessibility of microorganisms during the application of bactericidal rinsing solution in root canal treatments.

## Introduction

While analyzing the microbiota in endodontic lesions is a challenging task, it is necessary to enhance the success rate of endodontic therapy. Even though next-generation sequencing (NGS) methods have increased the number of species detected in root canals in studies over the past decade, the phyla and genera analyzed in primary as well as in secondarily infected root canals still show great variations between studies [1–6]. Because studies from the past decade still show great variations in regard to the composition and variety of microorganisms found, it is very challenging to develop new treatment strategies when one does not know the exact composition of microorganisms within endodontic lesions. To date, it is known that in primary apical periodontitis (PAP)-associated lesions, strictly anaerobic, proteolytic, and sometimes asaccharolytic species are predominant [7, 8], whereas secondary apical periodontitis (SAP) is mainly characterized by Gram-positive facultative anaerobes [7].

The great variety of results obtained from analyzing the microbiome in root canals over the past 10 years hinders the improvement of root canal treatment in general as the implementation of specific therapeutic strategies is complicated when one does not know the exact composition of bacteria in PAP and SAP-associated endodontic lesions. Therefore, there is a substantial need to improve and find new methods for the analysis of the microbiome of primary and secondary apical periodontitis associated lesions, especially because one possible explanation for the variation between studies might be due to different sampling procedures. At present, in vivo sampling or ex vivo cryo-pulverization are two well-known methods to extract bacterial DNA from infected root canals. In vivo sampling using sterile paper points is easy to conduct during endodontic treatment but bears the disadvantages of possibly mixing apical and coronal bacteria and the inability of gathering bacteria from apical ramifications. Ex vivo cryo-pulverization allows for the exclusive analysis of the apical part of the root canal system. This method is only applied on extracted teeth with severely infected endodontic lesions when conservative endodontic treatment is impossible [8].

In this study, we evaluated the impact of passive ultrasonic irrigation (PUI) on the in vivo sampling procedure using sterile paper points. PUI is to be considered a useful adjuvant in endodontic therapy as there is evidence for improved elimination of debris, bacteria, and smear layer within the root canal when PUI is combined with NaOCl [9, 10]. Previous studies have investigated the diverse effects of PUI on tissues and liquids. Some indicated that PUI exerts a cleansing effect through the creation of microstreaming, which can lead to the disaggregation of bacteria [11, 12] and also improves debridement within the canal [13]. Based on these findings, we hypothesized that PUI would increase the number of microorganisms found in root canals when combined with a classical in vivo sampling procedure. Because the current success rates of endodontic retreatments are much lower compared to initial root canal therapy, we used SAP-associated endodontic lesions as samples [14] to increase our knowledge about the composition of microorganisms within these lesions.

Most studies that analyzed the etiology of primary and secondary endodontic infections did not consider ultrasonic irrigation for the sampling procedure [1, 3, 4]. Hence, the present study sought to evaluate the impact of PUI on the in vivo sampling procedure and its effect on the bacteria, by analyzing the diversity and quantity of microorganisms within SAP-associated lesions.

## Materials and methods

### Patient selection and clinical parameters

Nineteen patients (four males, 15 females) with at least one persistent or recurrent, acute, or chronic apical lesion were involved in this study to examine the microbiota within the correspondent root canals. Patient selection was conducted at the Khartoum Centre for Research and Medical Training in North Sudan. All individuals showed good general health and had not received any antibiotic therapy within the last 30 days. They did not take part in another study during the same period. The women were neither pregnant nor breastfeeding. The age of the individuals was between 15 and 72 years. Before treatment anamnesis, the date, name, age, gender, tooth treated, age of root canal filling, and the condition of the cavity filling as well as the pain symptoms were recorded. Additionally, conventional single tooth imaging was performed prior to therapy to evaluate the depth and quality of the root filling and to exclude teeth with apical lesions of the periodontal origin or those that could not be properly prepared by instrumentation until 2 mm up to the apex.

All teeth included in this study had to have a root canal filling with radiological or clinical symptoms of an acute or chronic periodontitis apicalis. Improvement of the clinical situation by means of the therapy and by working under sterile conditions had to be feasible. Only single-root canal teeth were included, of which four displayed pain symptoms, and the rest showed apical radiolucency or a thickened desmodontal gap. Anterior teeth were used in the present study as they show less variation in canal anatomy and are therefore easier to compare. Detailed information of the patients is shown in Table 1.

**Table 1:** Patient characteristics

Patient	Gender	Age at treatment	Day of treatment	Symptoms	Tooth treated	Condition of cover filling	Primary endodontic treatment	X-ray finding
1	Female	56.2	21 March 2012	None	21	Insufficient	> 3 years	Root canal filling does not reach apical third
2	Female	21.9	22 March 2012	None	11	Insufficient	> 5 years	Leaky root canal filling; extended periodontal gap
3	Female	55.8	28 March 2012	Pain	23	Not evaluated	> 20 years	Root canal filling too long; minimal apical radiolucency
4	Male	41.5	29 March 2012	None	24 (2 canals)	Sufficient	> 2 years	Root canal filling too short
5	Female	41	31 March 2012	None	11 or 21	Not evaluated	> 8 years	Root canal filling insufficient; minimal apical radiolucency
6	Male	60.3	31 March n2012	None	45	Insufficient	> 8 years	Pronounced apical radiolucency
7	Male	31.4	04 April 2012	None	11	Sufficient	> 4 years	Insufficient mechanical debridement
8	Female	25.8	04 April 2012	None	15 or 25	Sufficient	> 5 years	Minimal apical radiolucency
9	Female	35.5	05 April 2012	Pain	22	Sufficient	> 4 months	Insufficient mechanical debridement; root canal filling not well adapted; apical radiolucency; crown
10	Female	17.4	09 April 2012	None	11	Sufficient	> 4 years	Apical radiolucency
11	Female	16	10 April 2012	None	25	Sufficient	> 2 months	Root filling not well adapted; apical radiolucency
12	Female	65	11 April 2012	Pain	21 or 11	Insufficient	> 2 months	11 and 21 both with root canal filling; root canal filling of 11 too short; 11 and 21 with apical radiolucency
13	Female	25.9	16 April 2021	None	34	Not evaluated	> 2 years ago	Root canal filling not well adapted; apical radiolucency
14	Female	55.8	28 March 2012	None	15	Sufficient	> 9 years	Root canal filling too short and not well adapted; apical radiolucency
15	Female	52.4	16 April 2012	None	35 or 45	Sufficient	> 20 years ago	Root canal filling too short; extended periodontal gap
16	Female	50.3	17 April 2012	None	14	Sufficient	> 5 years	Root canal filling up to 3 mm until apex; extended periodontal gap
17	Female	72.3	18 April 2012	None	32	Sufficient	> 1 year ago	Apical radiolucency
18	Female	72.3	18 April 2012	None	34	Sufficient	> 1 year ago	Apical radiolucency; crown
19	Male	17.1	20 March 2012	None	11	Sufficient	> 4 years	Root canal filling not well adapted and too short; apical radiolucency

The study design and all study protocols were reviewed and approved by the Ethical Committee of the Albert-Ludwigs-University of Freiburg (140/09) and the Ethical Committee of the El Razi College for Medical and Technological Sciences (Ref. KCRMT/ Nov 2011). Written informed consent was obtained from all patients.

### Sample collection

Microbiological samples were taken from the respective root canals before and after PUI using sterile paper points (ISO 25). To ensure sterile working conditions, a rubber dam was applied beforehand, followed by disinfection with 30% H_2_O_2_ and 3% NaOCl. Na_2_S_2_O_3_ was used to neutralize NaOCl to prevent unwanted side effects. To ensure proper decontamination, quality control (QC) samples of the enamel and dentine were taken using foam pellets. Insufficient root canal filling was removed by mechanical instrumentation only and root canals were instrumented up to size ISO 35, 2 mm short of the apex. An amount of 40 µl of 0.9% NaCl was applied into the root canal using sterile blunt needles (NaviTips, Ultradent, South Jordan, UT, USA; gauge 30), and sterile files were used for blending of the canal content. The samples prior to PUI (S1 samples, control group) were collected using three sterile paper points and transferred into vials containing 0.75 ml reduced transport fluid (RTF) [15]. Again, 40 µl NaCl was applied into the root canal and passive ultrasonic irrigation was conducted using a Handy Sonic UR-20P (Tomy Seiko., Ltd., Tokyo, Japan) and an ultrasound attachment (diameter 2.5 mm) for 3 min at a frequency of 28 kHz. Fresh NaCl was added every minute. The S2 sample (after PUI) was taken by inserting three sterile paper points up to 2 mm short of the apex and also transferred into a 0.75-ml RTF medium. S1 samples (after root filling removal, before PUI) served as a control group since these samples delivered the baseline values of microorganisms isolated without using PUI. The use of completely different teeth as a control group is not rational from a microbiological point of view, since the microbial composition of each infected tooth may be different.

Both S1 and S2 samples were stored at − 80 °C until further use.

### Preparation of samples

To enable quantitative analysis of the microbiota in the root canal samples and the identification of bacterial species, serial dilutions of 10^−3^, 10^−4^, and 10^−5^ for the S1, S2, and QC samples of each patient were prepared by diluting sample material in the peptone-yeast medium. Every dilution stage was plated onto three different culture media. Columbia Blood Agar plates (CoBI) were incubated for 5 days at 36 °C and 5–10% CO_2_ for the cultivation of aerobic species. Enterococci-selective agar plates were incubated under the same conditions for 2–3 days. Yeast-extract-cysteine blood agar plates (HCB) were incubated under anaerobic conditions (5% CO_2_, 85% N_2_, 10% H_2_) for 10 days at 36 °C to isolate anaerobic species. GENbox anaer-generators (bioMérieux, Marcy l’Etoile, France) ensured oxygen absorption, which was verified by anaerotest-indicator strips (Merck, Darmstadt, Germany).

### Isolation and differentiation of pure cultures

Isolation of pure cultures was achieved by phenotypical differentiation of colonies by means of shape, color, texture, size and hemolysis behavior, and quantitative registration of total colony number per ml (total colony number/ml = colony number × dilution degree). For the exact identification of different isolates, pure cultures were subcultivated on CoBI plates for aerobic and HCB plates for anaerobic isolates. Aerobic pure cultures were further differentiated by Gram stain, catalase, and oxidase testing. Anaerobic pure cultures were also differentiated by Gram stain as well as CO_2_ control plates and spot-indole testing.

For exact identification, three different methods were applied: MALDI-TOF MS (Microflex LT, Bruker Daltonik, Billerica, USA), the VITEK 2 system (bioMérieux, Marcy l’Etoile, France), and PCR amplification of the 16 s-rDNA with subsequent sequencing analysis.

### MALDI-TOF MS

MALDI-TOF MS was conducted as described in detail earlier [16]. In brief, a thin layer of pure culture was spread onto a sample carrier. Formic acid (1 µl of a 70% solution) was added, followed by drying and subsequent application of 1 µl of matrix solution at RT. Ionization and acceleration of agent macromolecules embedded in small matrix molecules in an electromagnetic field of 10–30 kV allowed detection of different times of flights and consequent database matching with 3740 reference spectra using the Biotyper software 3.0. The resulting similarity value was expressed as a log score ranging from ≥ 2.000 for identification on the species level to ≥ 1.700 for identification on the genus level.

### VITEK 2 system

If the results of the MALDI-TOF MS were uncertain, the VITEK 2 system was applied for further differentiation of pure cultures. The VITEK 2 system enables automatic identification and resistance testing of bacteria by analysis of different biochemical reactions using specific biochemical substrates and consequent database matching. For the examination of aerobic isolates, samples were mixed with 2.5 ml of 0.45% NaCl, and a McFarland turbidity of 0.5 was adjusted by means of photometric verification by DensiCHEK Plus (bioMérieux, Marcy l’Etoile, France). Anaerobic isolates were mixed with 3 ml 0.45% NaCl, and a McFarland turbidity of 2.70–3.30 was adjusted. For Gram-positive and Gram-negative bacteria, different test cards were used. Results were obtained 6 to 8 h after testing and rated with a probability index for a chemical reaction profile within one species.

### DNA extraction

If the identification of a species was still unsuccessful, the 16SrDNA-PCR method was applied. DNA extraction was achieved using two different methods. The first method was the quick DNA isolation after Reischl, whereby a single colony of a pure culture was extracted and transferred to a 1.5-ml Safe-Lock tube (Eppendorf, Hamburg, Germany) with 60 µl phosphate-buffered saline (PBS), followed by the addition of 240-µl lysis buffer (Reischl buffer) and homogenization by vortexing for 10 s. DNA release was performed by boiling at 99 °C while agitating at 700 rpm for 15 min. Samples were centrifuged at 18,400 g for 5 min, and the supernatant was transferred into a new 1.5-ml Safe-Lock tube and stored at 2–8 °C. If the Reischl method did not yield enough DNA, DNA extraction was achieved using the QIAamp DNA Mini Kit (QIAGEN GmbH, Hilden, Germany) according to the manufacturer’s instructions.

### 16Sr DNA-PCR

Bacterial 16S rDNA genes were amplified using the TP16U1 forward primer (5′-AGAGTTTGATC[C/A]TGGCTCAG-3′) and RT16U6 reverse primer (5′-ATTGTAGCACGTGTGT[C/A]GCCC-3′). PCR amplification was performed in a total volume of 50 µl. The reaction mixture contained 2 U of the HotStarTaq polymerase (QIAGEN, Hilden, Germany), 1 × PCR buffer (Tris–Cl, KCl, (NH4)_2_SO_4_, 3 mM MgCl_2_) (QIAGEN, Hilden, Germany), 0.2 mM of each of the four deoxyribonucleoside triphosphates (dNTPs), 0.5 mM of the primers, and 5 µl of the extracted sample DNA.

The PCR cycling conditions consisted of an initial denaturation step at 94 °C for 2 min, followed by 30 cycles that included another denaturation step at 94 °C for 1 min, annealing at 55 °C for 1 min, and extension at 72 °C for 1.5 min. The final extension occurred at 72 °C for 5 min. Positive and negative controls were included in each PCR reaction set. The PCR products were analyzed via gel-electrophoresis using the MCE-202 MultiNA-microchipelectrophoresis system (Shimadzu Corporation, Kyoto, Japan) following the manufacturer’s protocol.

### Sequencing of PCR products

Prior to sequencing, purification of PCR products using the GFX PCR DNA and Gel Band Purification Kit (GE Healthcare, Buckinghamshire, England) was performed. Cyclic sequencing was done using the Sanger method via BigDye®Terminator Kit v1.1 Cycle Sequencing Kit (Applied Biosystems, Darmstadt, Germany). For the reaction, 10.9-µl aqua bidest, 4.0 µl BigDye Terminator Seq. RR-100 (ddNTP), 0.1 µl BigDye Sequencing Buffer, and 3.0 µl DNA (PCR product) were mixed in a PCR Safe-Lock tube. The Sanger sequencing cycle conditions consisted of 25 cycles in total, starting with an initial denaturation at 98 °C for 1 min, annealing at 58 °C for 15 min, and extension at 60 °C for 240 min.

Precipitation and sedimentation of DNA followed. The precipitating agent consisted of 250 µl pure ethanol, 80 µl ultrapure water, and 10 µl 3 M NaAc (pH 5.2). After the addition of 20 µl cycle-sequencing product and homogenization for 20 s, the mix was centrifuged for 15 min at 15,870 g, and the supernatant was removed. Subsequently, 250 µl of 70% ethanol was added, followed by centrifugation at 15,870 g for 5 min, and removal of the supernatant. After drying of the sediment by vacuum centrifugation (Hetovac, Heto Holten A/S, Allerød, Denmark) for 5 min, 20 µl of Hi-Di™-formamide (Applied Biosystems, Darmstadt, Germany) was added, and the mix was pipetted into a sequencing tube.

Capillary electrophoresis was conducted using the ABI PRISM® 310 Genetic Analyzer (Applied Biosystems, Darmstadt, Germany). The fluorescence signal was detected by a charged coupled device camera and transferred onto an electropherogram. Chromatograms were checked for quality and changed manually if necessary. The 16S rDNA sequences were compared to those from the database of the National Center for Biotechnology Information (http://www.ncbi.nlm.nih.gov/BLAST). Sequences that showed 98% similarity or more were considered to be successfully identified.

### Statistical analysis

For descriptive analysis, mean values, standard deviations, and relative and absolute frequencies were computed. Bar charts were used for graphical presentation of the results and the Wilcoxon matched-pairs signed-rank test was used to test for differences between the samples. For the analyses, the statistics program Stata (StataCorp LT, College Station, TX, USA, version16.1) was used. The level of significance was set to 0.05.

## Results

### Total count of bacteria increased after passive ultrasonic irrigation (PUI)

**Fig. 1:**
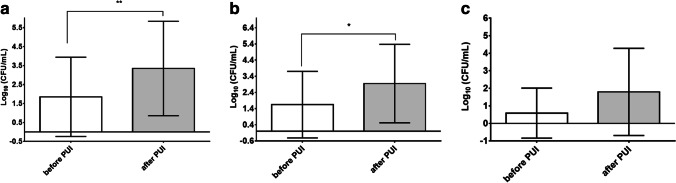
Count of microorganisms before and after PUI. **a** Total count of bacteria before (mean value log_10_: 4.28 CFU/ml) and after (mean value log10: 5.36 CFU/ml) PUI (**p < 0.01). **b** Aerobic/facultative anaerobic bacteria before (mean value log_10_: 4.21 CFU/ml) and after (mean value log_10_: 5.18 CFU/ml) PUI (*p < 0.05). **c** Obligate anaerobic bacteria before (mean value log10: 3.45 × 10^0^ CFU/ml) and after (mean value log_10_: 5.45 CFU/ml) PUI

As shown in Fig. 1a, the total count of bacteria ranged from 0 to 5.30 log_10_ CFU/mL before PUI (S1) (mean ± SD: 1.85 ± 2.09 log_10_ CFU/mL). After PUI (S2), the total bacterial count increased significantly (*p* < 0.01) and ranged from 0 to 6.71 log_10_ CFU/mL (mean ± SD: 3.35 ± 2.49 log_10_ CFU/mL) (Table 2). The high standard deviation was caused by non-detected bacteria in some samples before and after PUI. In 13 out of 19 probes, the number of the total bacterial count was higher after PUI than before.

**Table 2:** Bacterial counts detected in all patients before and after passive ultrasonic irrigation (PUI)

Patient	Total bacterial count in log_10_		Aerobic/facultative anaerobic bacteria in log_10_		Anaerobic bacteria in log_10_	
	Before PUI	After PUI	Before PUI	After PUI	Before PUI	After PUI
1	3.60	6.71	3.60	5.49	0.00	6.68
2	4.97	5.70	4.64	5.70	4.70	0.00
3	4.00	4.70	4.00	4.70	0.00	0.00
4	5.30	5.81	5.30	5.85	0.00	4.60
5	3.00	4.00	3.00	3.70	0.00	3.70
6	0.00	3.48	0.00	3.48	0.00	0.00
7	3.00	0.00	3.00	0.00	0.00	0.00
8	0.00	3.95	0.00	3.95	0.00	0.00
9	0.00	0.00	0.00	0.00	0.00	0.00
10	0.00	5.06	0.00	4.15	0.00	5.00
11	0.00	0.00	0.00	0.00	0.00	0.00
12	3.60	0.00	3.00	0.00	3.48	0.00
13	0.00	5.61	0.00	4.76	0.00	5.55
14	0.00	0.00	0.00	0.00	0.00	0.00
15	0.00	4.00	0.00	0.00	0.00	4.00
16	0.00	0.00	0.00	0.00	0.00	0.00
17	3.00	4.97	0.00	4.73	3.00	4.60
18	0.00	3.48	0.00	3.48	0.00	0.00
19	4.70	6.06	4.70	6.06	0.00	0.00

### Total count of aerobes/facultative anaerobes and obligate anaerobes increased after PUI

Before using PUI, aerobic and facultative anaerobic microorganisms were detected in eight S1 samples. Across all S1 samples, a range of 0 to 5.30 log_10_ CFU/mL (mean ± SD: 1.64 ± 2.49 log_10_ CFU/mL) was observed (Fig. 1b). After PUI, aerobic and facultative bacteria could be detected in 12 S2 samples. The range of aerobic and facultative anaerobic microorganisms was 0 to 6.06 log_10_ CFU/mL (mean ± SD: 2.95 ± 2.49 log_10_ CFU/mL) after PUI, which corresponds to a significant increase (*p* < 0.05) as compared to the values before using PUI.

Obligate anaerobic microorganisms were only detected in three S1 samples before PUI, whereas they could be detected in seven S2 samples after PUI. The range of obligate anaerobic bacteria was 0 to 4.70 log_10_ CFU/mL (mean ± SD: 0.59 ± 1.43 log_10_ CFU/mL) before PUI and 0 to 6.68 log_10_ CFU/mL (mean ± SD: 1.80 ± 2.48 log_10_ CFU/mL) after PUI (Fig. 1c). The increase of the number of obligate anaerobic bacteria after PUI was statistically not significant (*p* > 0.5) It should be emphasized that in six S2 samples, obligate anaerobes were detected, whereas no obligate anaerobic bacteria could be cultivated from their corresponding S1 samples.

### Diversity of detected bacteria increased after application of PUI

**Fig. 2:**
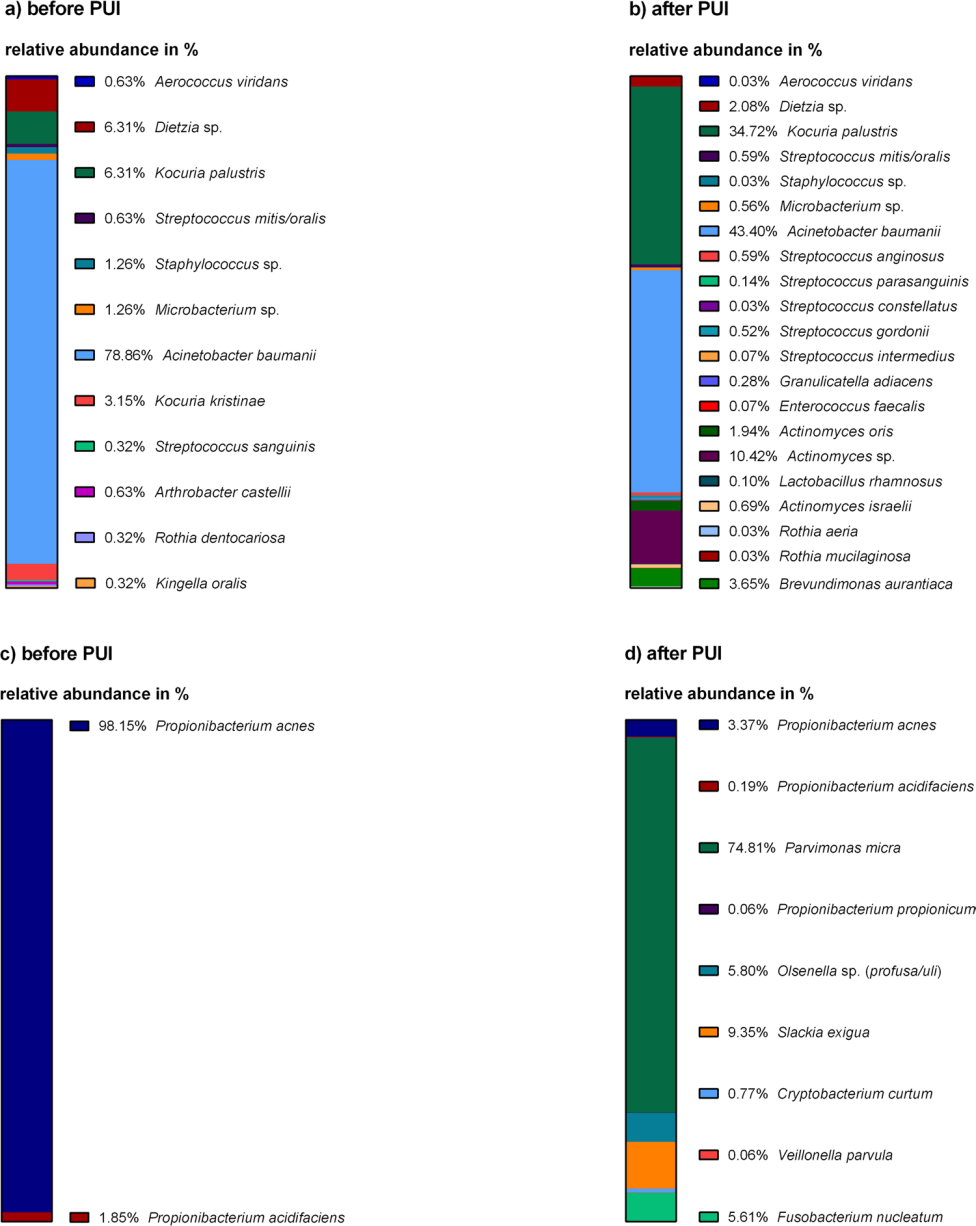
Detected aerobic/facultative anaerobic bacteria (relative abundance in %) before (**a**) and after (**b**) PUI. Detected obligate anaerobic bacteria (relative abundance in %) before (**c**) and after (**d**) PUI

Figure 2 displays all detected aerobic and facultative anaerobic bacteria (a and b) and all obligate anaerobic bacteria (c and d) before and after PUI. Considering aerobic and facultative anaerobic bacteria, the following could be observed: Before PUI, 12 different aerobic/facultative anaerobic bacteria could be detected (Fig. 2a), whereas this number increased up to 21 after PUI (Fig. 2b). Seven bacterial species were detected before and after PUI (*Aerococcus viridans, Dietzia* sp*., Kocuria palustris, Streptococcus mitis/oralis, Staphylococcus* sp*., Microbacterium* sp*., Acinetobacter baumanii*), five were exclusively detected before PUI (*Kocuria kristinae, Streptococcus sanguinis, Arthrobacter castellii, Rothia dentocariosa, Kingella oralis*), and 14 were exclusively detected after PUI (*Streptococcus anginosus, Streptococcus parasanguinis, Streptococcus constellatus, Streptococcus gordonii, Streptococcus intermedius, Granulicatella adiacens, Enterococcus faecalis, Actinomyces oris, Actinomyces* sp*., Lactobacillus rhamnosus, Actinomyces israelii, Rothia aeria, Rothia mucilaginosa, Brevundimonas aurantiaca*). In both sample types, *Acinetobacter baumanii* was the most commonly occurring microorganism.

Considering all obligate anaerobic bacteria detected before and after PUI, only two different bacterial species could be detected before PUI (Fig. 2c), namely *Propionibacterium acnes* (98.15%) and *Propionibacterium acidifaciens* (1.85%). After PUI (Fig. 2d), the number increased up to nine different bacterial species, namely *P. acnes*, *P. acidifaciens*, *Parvimonas micra*, *Propionibacterium propionicum*, *Olsenella* sp. *(profusa/uli)*, *Slackia exigua*, *Cryptobacterium curtum*, *Veillonella parvula*, and *Fusobacterium nucleatum*.

**Fig. 3:**
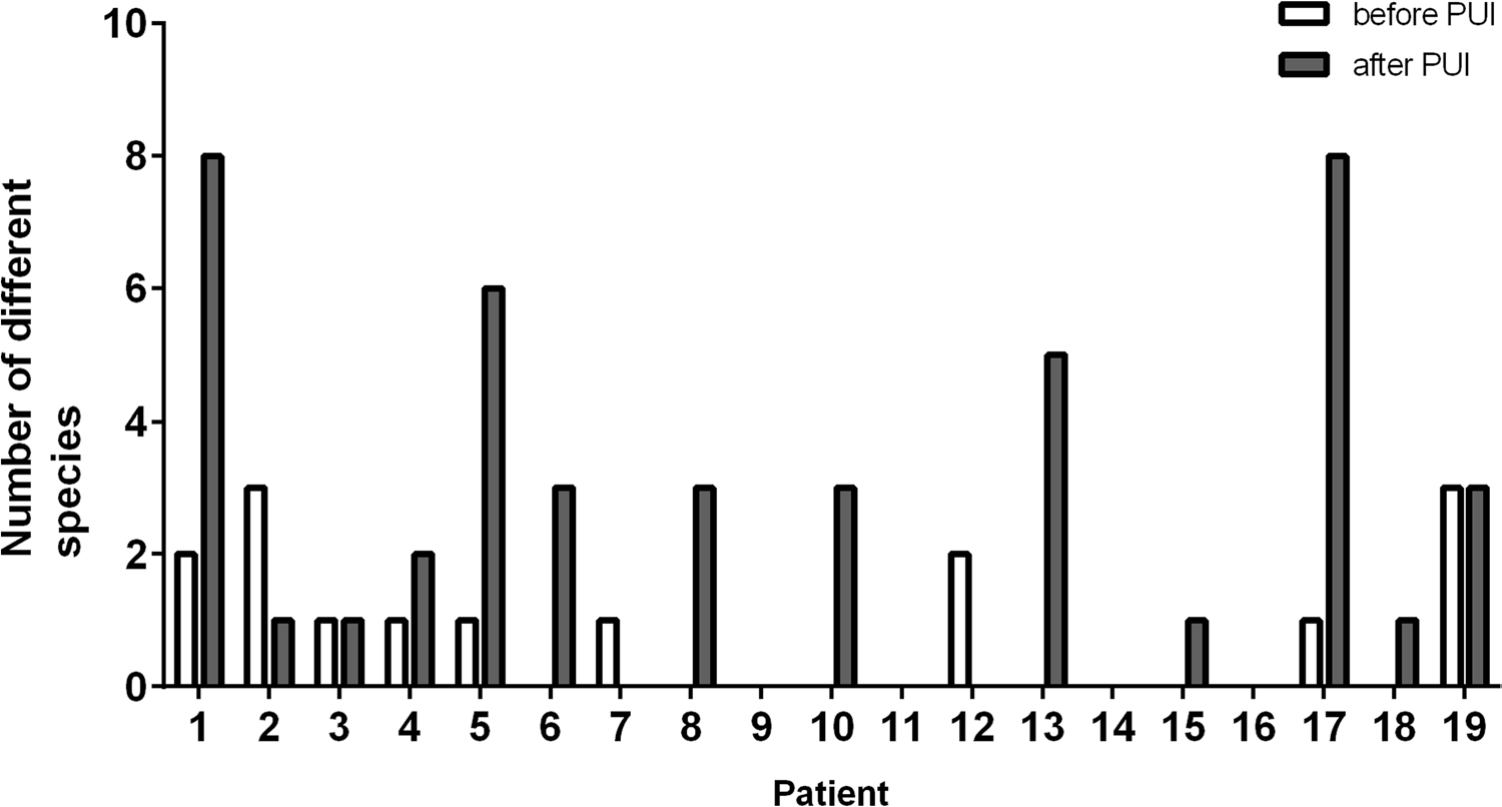
Number of different species detected per patient. Patients 1, 4, 5, 6, 8, 10, 13, 15, 17, and 18 displayed higher numbers of different species after PUI; patients 3 and 19 displayed equal numbers of different species before and after PUI; patients 2, 7, and 12 displayed lower numbers of different species after PUI; in patients 9, 11, 14, and 16, there was no detection of any species before and after PUI

Figure 3 shows the number of different species detected before and after PUI for every patient. In 10 out of 19 patients (patient 1, 4, 5, 6, 8, 10, 13, 15, 17, 19) the number of different species detected increased after PUI, while three patients displayed higher numbers of different species before PUI (patient 2, 7, 12), and in two patients, the number of species was the same before and after PUI (patient 3, 19). In four patients, no bacteria were detected both before and after PUI.

## Discussion

The aim of this study was to evaluate the impact of PUI to improve the quantity and diversity of the detected bacterial flora within secondary infected root canals. Sample collection was performed during endodontic retreatment using sterile paper points before (S1) and after PUI (S2). The sample size used in the present study was previously shown to be sufficient to reveal the microbial composition of infected root canals in similar studies [15, 17, 18].

A significant increase of the overall bacterial count after PUI was observed, as shown in Fig. 1a), indicating that PUI using NaCl as a solution exerts no bactericidal but rather a cleansing effect. This cleansing effect can be explained by an important biophysical phenomenon created by ultrasound, the so-called acoustic current or microstreaming effect. Acoustic current is defined as a time-independent, fast circulation of liquids in one direction formed either by obstacles within an acoustic field or the proximity of small vibrating objects [19]. It can lead to the disaggregation of bacteria [11, 12] and the improvement of debridement within root canals [13]. Disaggregation of biofilms within dentin tubuli is very advantageous as it allows a more precise analysis of microorganisms within root canals and enhances the sensitivity of bactericidal measures [11]. Another study also confirmed that PUI improves the penetration of cleansing solutions into apical ramifications and dentin tubuli [20]. Our results indicate that PUI could be a useful tool for the improvement of traditional in vivo sampling procedures using sterile paper points, as they are not able to collect bacteria from apical ramifications [2, 20]. Combining in vivo sampling procedures with PUI, as done in this study, significantly increased the number of bacteria detected in secondary infected root canals. This may indicate that PUI could help the removal of bacteria from apical ramifications and dentin tubuli that are difficult to access. To further verify this point, it would be interesting to compare the outcome of bacterial analysis when using an ex vivo sampling procedure such as cryo-pulverization to ensure that solely the apical part of the root canal system is examined. It should be emphasized that although better results were obtained from using PUI, the application of this technique may not have the same effects on areas that are difficult to reach such as fins, lateral canals, and isthmuses. Additionally, in curve root canals, the PUI device cannot be taken all the way down to working length, making it impossible to obtain all microorganisms and debris from the apical segment of the root canal.

Our results support the view that PUI alone exerts no bactericidal effect. Other studies investigated the phenomenon of transient cavitation, which is also created by ultrasound, and found that it can lead to the creation of shockwaves and radicals that can weaken or destroy bacteria [11]. If transient cavitation had taken place in our study, the total number of bacteria in S2 samples most likely would have decreased. Furthermore, many studies indicate that the lumen of root canals is too small to support the effect of transient cavitation and concluded that transient cavitation plays a tangential role when using PUI during endodontic treatment [21–23].

When using a bactericidal cleansing solution such as NaOCl as a medium for PUI, many studies show a lower abundance of bacteria after ultrasonic irrigation [24, 25]. This can be explained by the synergistic effects of PUI and NaOCl leading to an improved debridement of root canals, removal of smear layer, and tissue dissolution [24, 25].

Differentiating between aerobes/facultative anaerobes (Fig. 1b) and obligate anaerobes (Fig. 1c) showed that a significant increase of bacteria was detected in S2 samples only for aerobes/facultative anaerobes, even though the total number of strictly anaerobic bacteria was higher than the total number of facultative aerobes.

Overall aerobes/facultative anaerobes are more resistant to antimicrobial substances and more often expected to be found in root canal-treated teeth [26]. Out of 19 samples, 15 were positively identified as being aerobes/facultative anaerobes. The higher abundance of obligate anaerobes is caused by one specific sample (patient 1) that displayed extremely high counts of obligate anaerobes in the S2 sample (6.68 log_10_ CFU / mL), whereas the S1 sample was negatively evaluated. Before PUI, obligate anaerobes were only found in three samples, while in contrast, after PUI, seven samples displayed numbers of obligate anaerobes. As obligate anaerobes were found in both S1 and S2 samples in patient 17 only, it can be concluded that in most cases, PUI enabled the detection of obligate anaerobes in the first place, and that when it is not used, no obligate anaerobic microorganisms would have been found at all. To compare this, however, it should be mentioned that the diversity of obligate anaerobes detected was lower compared to that found in previous studies in which the percentage of obligate anaerobic pathogens made up over 40% of the number of detected microorganisms [27]. In our study, nine different anaerobic species were found, which counts for 27.3% of the total microbial spectrum represented.

The diversity of microorganisms also increased after PUI. Whereas in the S1 samples, only an average of 1.1 different species was found, the S2-samples displayed an average of 3.1 different species. This represents an approximately threefold increase in diversity. Previous studies detected an average of 1.3–1.8 different species in root canal-treated teeth [26, 27]. This indicates that the application of PUI substantially improves the detection of bacteria during endodontic revision. Furthermore, other studies using next-generation sequencing methods for the detection of bacteria demonstrate that species richness in endodontic revisions is much higher than anticipated in culture studies [28, 29]. Combining NGS methods and PUI could reveal that the number of different species in secondary endodontic infections is underestimated so far.

In this study, 33 different species could be identified, of which 12 were detected before and 29 after PUI use. The prevalence of positive cultures also increased after PUI: out of 60 positive cultures, 15 were derived from S1 samples and 45 from S2 samples. That means that not only the diversity but also the number of microorganisms found in secondary endodontic infections increases after using PUI. Thus, PUI facilitates the removal of certain pathogens that would not have been detected without its use. For example, aerobic/facultative anaerobic microorganisms such as *Actinomyces oris* (1.94%), *Actinomyces* sp. (10.42%), and *Brevundimonas aurantiaca* (3.65%), as well as obligate anaerobic bacteria such as *Parvimonas micra* (74.81%), *Slackia exigua* (9.35%), and *Fusobacterium nucleatum* (5.61%) showed a high relative abundance only after PUI.

The isolated flora predominantly comprised Gram-positive aerobes/facultative anaerobes, which is in line with other studies [7, 26]. Therapy of Gram-positive bacteria is difficult because of their robustness and their adaptability [26]. Previous studies describe a high prevalence of *Streptococcus* spp. and *Enterococcus* spp*.* in persistent apical periodontitis [26, 28]. Our results show that after PUI, the numbers of streptococci nearly doubled. One explanation for this could be the ability of streptococci to invade dentin tubuli [30] and that they are washed out due to the microstreaming phenomena caused by PUI.

*E. faecalis* was long thought to be one of the most common pathogens in secondary infected root canals, representing up to 47% of all germs found in total [27, 28, 31]. In contrast, we only found *E. faecalis* twice after PUI and in very low amounts (0.07%), barely meeting the detection threshold even though a selective medium was used for cultivation. Our results are in line with more recent studies questioning the importance of this pathogen in endodontic revisions [2, 4, 5, 17, 18]. Interestingly, some root canals bear high loads of *E. faecalis* despite the overall low prevalence [4, 5]. As secondary endodontic infections can either be caused by bacteria present before the initial treatment or bacteria invading the root canal following treatment, Manoil and colleagues hypothesized that *E. faecalis* might be a secondary invader as it is more abundant in root canals that were treated over several sessions or left open for some time [8]. This could explain the high load of *E. faecalis* in some root canals of secondary infected endodontic roots but an overall lesser prevalence than anticipated in older studies [8, 17, 32].

The percentage of Gram-positive rods, also commonly found in endodontic revisions [26, 33], greatly increased after PUI. Especially the exclusive evidence of *Actinomyces* spp*. (Actinomyces oris, Actinomyces israelii*, *and Actinomyces* spp*.)* and *Propionibacterium propionicum* after PUI should be noted, as they are often mentioned in the context of failed endodontic treatment [34]. Thus, PUI seems to be a helpful tool in the elimination of these pathogens from secondary infected root canals.

Regarding the microbial count, Gram-negative rods represented the second largest group after Gram-positive cocci with 22.17%. Out of that total, 17.45% accounted for *A. baumannii* showing a significant increase in abundance in S2 samples. Another study also reported high numbers of this pathogen in endodontic revisions, with a frequency of 69–88% [1]. *A. baumannii* is associated with multiple antibiotic resistances, high robustness, and the ability to survive for long periods under different environmental conditions [35]. Therefore, it can be concluded that PUI supports the elimination of very adaptable microorganisms in particular. Only a few Gram-negative cocci were found amongst the obligate anaerobes in this study, which is in line with previous results [26].

In general, a certain variability regarding the quantity and the diversity of microorganisms found in secondary infected root canals is to be expected and is due to inter and intra-individual differences and geographical origin [36]. The sampling technique and subsequent detection method can also cause variation in the quantity and diversity of microorganisms found. An increase of the sample size in future studies would mitigate the effects of variations and provide more supporting evidence for the benefit of using PUI in cleansing infected teeth during the endodontic treatment.

In conclusion, PUI is a powerful tool to improve the in vivo sampling technique using sterile paper points and therefore to enhance both the quantity and the diversity of microorganisms found in secondary endodontic infections. Combining PUI with NGS methods could substantially improve our understanding of the microflora involved in the pathogenesis of secondary infected root canals and may lead to the implementation of better treatment strategies.

Further, PUI causes microstreaming in vivo and thus is a valuable adjuvant for endodontic treatment. Besides the already known strengthening of the cleansing effect via debridement and removal of smear layer [37], this study also indicates that PUI may increase the dissolution of inaccessible and attached microorganisms within apical ramifications and dentin tubuli. To further verify this effect, additional ex vivo experiments using cryo-pulverization of the apical part of the root canal system would be useful. As PUI does not lead to the destruction of microorganisms, the additional use of antimicrobial cleansing solutions such as NaOCl during endodontic treatment is recommended.
